# Supplementary material for: Dynalign II: common secondary structure prediction for RNA homologs with domain insertions
Source: Nucleic Acids Res. 2014 Nov 21;42(22):13939–48. doi: 10.1093/nar/gku1172 (PMC4267632; doi:10.1093/nar/gku1172)
Supplement: SUPPLEMENTARY DATA [file supp_gku1172_nar-02021-z-2014-File012.zip › manual/Overview/Libraries.html]

RNAstructure Installation and Overview: Libraries


|  |  |  |
| --- | --- | --- |
|  | RNAstructure Installation and Overview Libraries | - Contents - Index |
| The Dynalign Library  The Dynalign library holds information necessary to build a Dynalign object, which is used for running Dynalign. This library is self-contained and depends on no other libraries once built. The names of this library, depending on the operating system on which it is built, are as follows:   - Linux systems, 32 or 64 bit: libDynalign.so - Macintosh systems: libDynalign.dylib - Windows systems (cygwin): Dynalign\_Library.dll  ---   The Dynalign SMP Library  The Dynalign SMP library holds information necessary to build a Dynalign object. This library is self-contained and depends on no other libraries once built. Unlike the Dynalign library (above), the Dynalign SMP library can be used in parallel SMP computing environments. The names of this library, depending on the operating system on which it is built, are as follows:   - Linux systems, 32 or 64 bit: libDynalign\_SMP.so - Macintosh systems: libDynalign\_SMP.dylib - Windows systems (cygwin): Dynalign\_SMP\_Library.dll  ---   The Hybrid RNA Library  The Hybrid RNA library holds information necessary to build a Hybrid RNA object, which is used for bimolecular secondary structure prediction. This library is self-contained and depends on no other libraries once built. The names of this library, depending on the operating system on which it is built, are as follows:   - Linux systems, 32 or 64 bit: libHybridRNA.so - Macintosh systems: libHybridRNA.dylib - Windows systems (cygwin): HybridRNA\_Library.dll  ---   The Multilign Library  The Multilign library holds information necessary to build a Multilign object, which is used for running Multilign. This library is self-contained and depends on no other libraries once built. The names of this library, depending on the operating system on which it is built, are as follows:   - Linux systems, 32 or 64 bit: libMultilign.so - Macintosh systems: libMultilign.dylib - Windows systems (cygwin): Multilign\_Library.dll  ---   The Multilign SMP Library  The Multilign SMP library holds information necessary to build a Multilign object, which is used for running Multilign. This library is self-contained and depends on no other libraries once built. Unlike the Multilign library (above), the Multilign SMP library can be used in parallel SMP computing environments. The names of this library, depending on the operating system on which it is built, are as follows:   - Linux systems, 32 or 64 bit: libMultilign\_SMP.so - Macintosh systems: libMultilign\_SMP.dylib - Windows systems (cygwin): Multilign\_SMP\_Library.dll  ---   The Oligo Library  The Oligo library holds information necessary to build an Oligowalk object, which is used for running OligoWalk and OligoScreen. This library is self-contained and depends on no other libraries once built. The names of this library, depending on the operating system on which it is built, are as follows:   - Linux systems, 32 or 64 bit: libOligo.so - Macintosh systems: libOligo.dylib - Windows systems (cygwin): Oligo\_Library.dll  ---   The PARTS Library  The PARTS library holds information necessary to build a t\_ppf\_loops object, which runs PARTS calculations. This library is not self-contained, and must be linked together with the RNA library into an executable. The names of this library, depending on the operating system on which it is built, are as follows:   - Linux systems, 32 or 64 bit: libPARTS.so - Macintosh systems: libPARTS.dylib - Windows systems (cygwin): PARTS\_Library.dll  ---   The Postscript Library  The Postscript library holds information necessary to build a variety of specific objects, each of which has the ability to draw a specific type of Postscript image. This library is not self-contained, and must be linked together with the Dynalign library into an executable. The names of this library, depending on the operating system on which it is built, are as follows:   - Linux systems, 32 or 64 bit: libPS.so - Macintosh systems: libPS.dylib - Windows systems (cygwin): Postscript\_Library.dll  ---   The RNA Library  The RNA library holds information necessary to build an RNA object, which is a basic representation of a single strand of nucleic acids and any data associated with it. This library provides access to most single sequence secondary structure prediction and analysis methods. This library is self-contained and depends on no other libraries once built. The names of this library, depending on the operating system on which it is built, are as follows:   - Linux systems, 32 or 64 bit: libRNA.so - Macintosh systems: libRNA.dylib - Windows systems (cygwin): RNA\_Library.dll  ---   The TurboFold Library  The TurboFold library holds information necessary to build a TurboFold object, which is used for running TurboFold. This library is not self-contained, and must be linked together with the RNA library into an executable. The names of this library, depending on the operating system on which it is built, are as follows:   - Linux systems, 32 or 64 bit: libTurboFold.so - Macintosh systems: libTurboFold.dylib - Windows systems (cygwin): TurboFold\_Library.dll  ---   The TurboFold SMP Library  The TurboFold SMP library holds information necessary to build a TurboFold object, which is used for running TurboFold. This library is not self-contained, and must be linked together with the RNA library into an executable. Unlike the TurboFold library (above), the TurboFold SMP library can be used in parallel SMP computing environments. The names of this library, depending on the operating system on which it is built, are as follows:   - Linux systems, 32 or 64 bit: libTurboFold\_SMP.so - Macintosh systems: libTurboFold\_SMP.dylib - Windows systems (cygwin): TurboFold\_SMP\_Library.dll  ---   The RNAstructure (JAVA) GUI Library  The RNAstructure (JAVA) GUI library, unlike the other libraries on this page, is a dynamic library, so it must always stay in the RNAstructure/exe directory, and is not capable of being used for anything other than its connection to the RNAstructure GUI. It holds all information necessary to run calculations from the RNAstructure GUI, so it contains some elements of all the other libraries on this page. This library is self-contained and depends on no other libraries once built. The names of this library, depending on the operating system on which it is built, are as follows:   - Linux systems, 32 or 64 bit: libRNAstructure\_GUI.so - Macintosh systems: libRNAstructure\_GUI.dylib - Windows systems (cygwin): RNAstructure\_GUI.dll | | |
| Visit The Mathews Lab RNAstructure Page for updates and latest information. | | |
